# Retraction: Designing of SiO_2_ mesoporous nanoparticles loaded with mometasone furoate for potential nasal drug delivery: *Ex vivo* evaluation and determination of pro-inflammatory interferon and interleukin mRNA expression

**DOI:** 10.3389/fcell.2024.1540228

**Published:** 2024-12-13

**Authors:** Frontiers Editorial Office

The journal retracts the 6 January, 2023 article cited above.

Following publication, concerns were raised regarding the integrity of the images in this article. An investigation was conducted in accordance with Frontiers’ policies.

It was found that the concerns were valid and that the article does not meet the standards of editorial and scientific soundness for Frontiers in Cell and Developmental Biology; therefore, the article has been retracted.

The retraction was approved by the Chief Editors for Frontiers in Cell and Developmental Biology and the Chief Executive Editor of Frontiers. The authors do not approve of the retraction.

